# Pretargeted Imaging and Radioimmunotherapy of Cancer Using Antibodies and Bioorthogonal Chemistry

**DOI:** 10.3389/fmed.2014.00044

**Published:** 2014-11-18

**Authors:** Floor C. J. van de Watering, Mark Rijpkema, Marc Robillard, Wim J. G. Oyen, Otto C. Boerman

**Affiliations:** ^1^Department of Radiology and Nuclear Medicine, Radboud University Medical Center, Nijmegen, Netherlands; ^2^Tagworks Pharmaceuticals, Eindhoven, Netherlands

**Keywords:** pretargeting, bispecific antibodies, tumor-associated antigen, radioimmunodetection, radioimmunotherapy

## Abstract

Selective delivery of radionuclides to tumors may be accomplished using a two-step approach, in which in the first step the tumor is pretargeted with an unlabeled antibody construct and in the second step the tumor is targeted with a radiolabeled small molecule. This results in a more rapid clearance of the radioactivity from normal tissues due to the fast pharmacokinetics of the small molecule as compared to antibodies. In the last decade, several pretargeting approaches have been tested, which have shown improved tumor-to-background ratios and thus improved imaging and therapy as compared to directly labeled antibodies. In this review, we will discuss the strategies and applications in (pre-)clinical studies of pretargeting concepts based on the use of bispecific antibodies, which are capable of binding to both a target antigen and a radiolabeled peptide. So far, three generations of the bispecific antibody-based pretargeting approach have been studied. The first clinical studies have shown the feasibility and potential for these pretargeting systems to detect and treat tumor lesions. However, to fully integrate the pretargeting approach in clinic, further research should focus on the best regime and pretargeting protocol. Additionally, recent developments in the use of bioorthogonal chemistry for pretargeting of tumors suggest that this chemical pretargeting approach is an attractive alternative strategy for the detection and treatment of tumor lesions.

## Introduction

**Figure 1:**
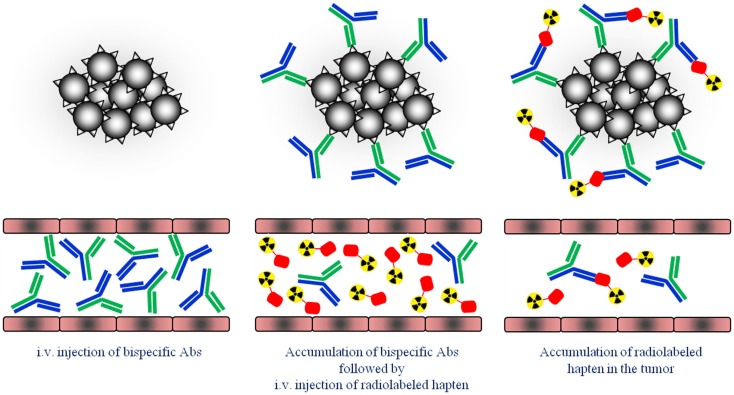
**Schematic overview of the pretargeting strategy**. First, tumors are pretargeted with bispecific antibodies (bsAb). Secondly, small radiolabeled hapten peptide is i.v. injected and binds to the pretargeted bsAb at the tumor cells.

The use of radiolabeled monoclonal antibodies (mAbs) directed against tumor-associated antigens to visualize, characterize, and/or treat tumor lesions has been widely studied in preclinical and clinical studies (1–3). After intravenous injection, the accumulation of the radiolabeled mAbs in the tumor is slow due to the slow extravasation and tissue penetration of intact antibodies. Various physiological barriers between the circulation and the tumor cell surface, such as the vascular endothelium, the relatively large transport distances, and the high interstitial fluid pressure in tumors, prevent the rapid accretion of the antibodies in the tumor (4). As a result, only a small fraction of the injected activity will localize in the tumor. Despite this inefficient targeting, in various clinical settings this approach can be effective: especially radiosensitive tumors, like Non-Hodgkin’s lymphomas, can respond to treatment with radiolabeled anti-CD20 antibodies (5). Effective radioimmunotherapy (RIT) requires high tumor uptake and rapid clearance of radioactivity from normal tissues. Various approaches have been investigated to improve the tumor targeting such as reducing the circulatory half-life of the antibodies (4). Molecular engineering techniques made it possible to produce antibody formats that clear faster from the blood. However, this is the central dilemma in antibody targeting of tumors: on one hand, the high level of mAb in the circulation is the driving force for the accumulation of the mAb in the tumor; on the other hand, the clearance from normal tissues should be rapid. Therefore, innovative approaches are needed to enhance tumor accumulation while limiting retention in normal tissues. Pretargeting is an approach to solve this problem. Several pretargeting systems have been developed including the avidin/streptavidin-biotin pretargeting system (4, 6) exploiting the extremely high binding affinity between biotin and (strept)avidin (*K*_d_ = 4 × 10^−14^ M) (7), the DNA-complementary DNA binding pretargeting system, which relies on the high affinity interaction between complementary oligomers (8–11), pretargeting systems based on the use of bispecific antibodies (bsAb), and recently a novel pretargeting approach has been reported based on highly selective bioorthogonal chemical reactions (12, 13). All pretargeting systems are promising; however, each of these approaches has its advantages and limitations. For the avidin/streptavidin, the most significant limitation is the immunogenicity of the pretargeting agents: (strept)avidin being a protein that cannot be humanized. Additionally, the presence of endogenous biotin that could interfere with the system and the need to use a clearing agent to remove the residual antibody from the circulation before administration of the radiolabeled biotin limit the application of this system (14). For the DNA-complementary DNA binding pretargeting system, no immunogenic issues or complications due to the presence of competing endogenous species are reported. However, the oligonucleotides need to be modified to prevent rapid degradation by DNases and/or RNases (11). The pretargeting concept based on the use of bsAb directed against both a target antigen and a radiolabeled hapten is carried out in the following two steps. In the first step, the bsAb is injected. After the bsAb has accumulated in the tumor due to the specific binding to the tumor-associated antigen and cleared from the blood, the radiolabeled peptide carrying the hapten is administrated. This small molecule will be trapped in the tumor by binding to the bsAb or cleared rapidly from the body via the kidneys. A schematic overview of this pretargeting strategy is shown in Figure 1. Depending on the radionuclide used this pretargeting approach can be used to visualize, or treat tumor lesions, for example, using ^111^In and ^99m^Tc for single-photon emission computed tomography (SPECT) imaging, ^68^Ga or ^18^F for positron emission tomography (PET) imaging, or ^131^I, ^90^Y, and ^177^Lu for pretargeted radioimmunotherapy (PRIT). A significant advantage of this system over the biotin/avidin-based approaches is that the primary pretargeting agent, the bsAb, can be humanized to reduce its immunogenicity. Additionally, the lower affinity of the bsAb-hapten binding compared to the avidin-biotin binding results in the rapid dissociation of the bsAb from the hapten in the circulation, thereby omitting the need for a clearing agent. In the past decade, several improvements on this system have been introduced, which resulted in a flexible universal and efficient pretargeting system. However, the non-covalent binding between the radiolabeled hapten and the bispecific antibody at the tumor cell surface limit the retention of the radiolabeled hapten-peptide in the tumor. Recent developments in the use of bioorthogonal chemistry (cf. the rapid and selective formation of a covalent bond between the pretargeting agent and the radiolabeled agent *in vivo*) are very promising and show the potential of this system for efficient pretargeting of tumors. Thus, this chemical pretargeting system is an attractive alternative for bsAb-based pretargeting.

In this review, we will focus on the pretargeting concept based on the use of bsAb and additionally discuss the potential and the limitations of this novel bioorthogonal chemistry pretargeting approach.

## Bispecific Antibody-Based Pretargeting Systems

The first studies on the use of a two-step system to target tumors were described by Reardan et al. (15). This group used antibodies against radiolabeled EDTA. This study showed that by separating the antibody tumor targeting from radionuclide targeting improved tumor-to-non-tumor ratios could be obtained (16). These antibodies had no affinity to the tumor and accumulated passively due to the enhanced permeability and retention effect in the tumor (4, 17, 18). For specific pretargeting of tumors, bsAb directed against both the tumor-associated antigens and the radiolabeled agent are required.

### First generation; anti-tumor Fab′ × anti-chelate-metal Fab′ fragments

The first generation of bsAb for specific pretargeting methods was chemically conjugated anti-tumor Fab′ × anti-chelate-metal Fab′ fragments (6). Several preclinical studies revealed good tumor uptake (19, 20). Stickney et al. linked the sulfhydryl groups of Fab′ fragments of anti-CEA to those of an anti-benzyl-EDTA to form an F(ab′)_2_ bifunctional antibody (19). The potential of this anti-CEA × anti-benzyl-EDTA F(ab′)_2_ was evaluated in mice bearing colon tumor xenografts. The anti-CEA × anti-benzyl-EDTA Fab′ × Fab′was injected 24 h prior to the i.v. administration of ^111^In-labeled benzyl-EDTA. One day later, the biodistribution revealed a tumor uptake of 18.5%ID/g, whereas the blood level was 1.3%ID/g. A clinical study was performed to assess the use of this approach in patients with recurrent colon carcinoma. In 14 patients, 20 of the 21 known tumor lesions were detected by scintigraphic imaging. Additionally, nine unknown lesions were detected of which eight could be confirmed.

### Second generation; the use of a divalent radiolabeled hapten-peptide

The pretargeting system using anti-tumor Fab′ × anti-chelate-metal Fab′ fragments was based on monovalent binding to the chelate–radiometal complex at the tumor. The stability of such a complex is limited and rapid release of the radiolabeled chelate from the tumor may occur (21). Le Dousall et al. reported improved stability of the complex using divalent hapten-peptide (22). In BALB/c mice i.v. injection of bsAb directed against the lyb8.2 antigen and the hapten dinitrophenyl (DNP) followed by either the mono [*N*-ε-(2,4-dinitropheny1)-l-lysyl-diethylenetriaminepentaacetic acid (DNP-DTPA)] or divalent (di-DNP-DTPA).

**Figure 2:**
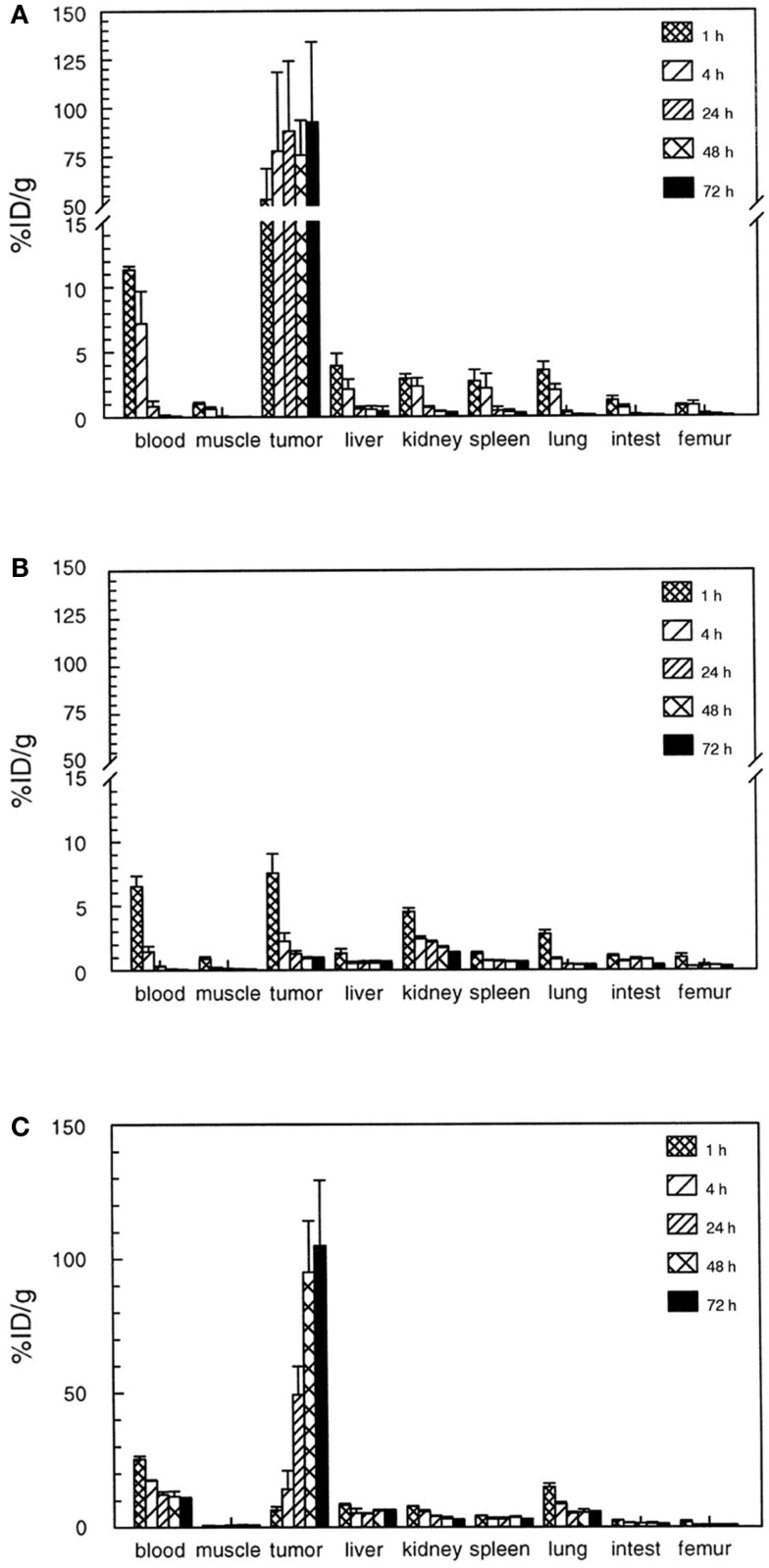
**Biodistribution of 111In-diDTPA (A) and 111In-DTPA (B) at 1?72 h after injection of the radioactivity in nude mice with s.c. NU-12 tumors**. Mice received 100 pmol of G250 × DTIn1 bsAb i.v. and 3 days later 7 pmol of 111In-DTPA or 111In-diDTPA. As a reference a separate set of nude mice with s.c. NU-12 xenografts received the directly labeled antibody 111In-DTPA-G250 **(C)**. This research was originally published in Cancer Research by Boerman et al. (16).

The ^125^I-divalent DNP derivative exhibited a significantly higher binding as compared to the monovalent derivative to the pretargeted cells (for monovalent 23 vs. 65%ID/g for the divalent derivative). It was hypothesized that at the cell surface the divalent hapten could cross-link two bsAbs thereby stabilizing not only the binding to the bsAb but also the bsAb binding to the tumor. This so-called affinity enhancement system (AES) improved tumor targeting and retention of the radiolabeled di-hapten-peptide (20, 22). This AES was confirmed by different groups in several animal models, in which enhanced tumor uptake and improved retention of the radiolabeled di-hapten-peptide was observed (23–28). For example, the use of a bsAb in combination with a divalent ^111^In-DTPA-peptide resulted in a tumor uptake of 3.5%ID/g whereas the tumor uptake of the monovalent DTPA was 2.8%ID/g in mice with s.c. A375 melanoma xenograft. A pronounced AES effect was observed in nude mice with RCC xenografts using bsAb directed against the renal cell carcinoma-associated antigen G250, in combination with a divalent hapten-peptide, Phe–Lys(DTPA-^111^In)–Tyr–Lys(DTPA-^111^In), ^111^In-di-DTPA (16). At 1 h p.i. of the hapten, an almost 10-fold higher tumor uptake of ^111^In-di-DTPA (80%ID/g) was observed as compared to the monovalent ^111^In-DTPA (9%ID/g; Figure 2). Moreover, the retention of the radiolabel in the tumor at 72 h p.i. was significantly higher for the ^111^In-di-DTPA (93 ± 41%ID/g) compared to ^111^In-DTPA (0.9 ± 0.1%ID/g).

Based on the promising preclinical studies, a clinical trial was performed to evaluate the pretargeting approach in patients with primary colorectal cancers. In 11 patients with primary colorectal carcinoma tumors, anti-CEA × anti-DTPA Fab′–Fab′ antibody was injected followed 2–8 days later by ^111^In-labeled *N*-α-(In-DTPA)-tyrosyl-*N*-ε-(In-DTPA)-lysin (^111^In-di-DTPA) (29). For comparison, six patients with similar clinical status were injected with ^111^In-anti-CEA F(ab′)_2_. At 1–4 days p.i. of ^111^In-di-DTPA, the biodistribution results revealed a tumor uptake of 1.8–17.5%ID/kg and were similar to the tumor uptake of ^111^In-anti-CEA F(ab′)_2_ (5.5–30.2%ID/kg). However, the tumor-to-blood (T/B) and the tumor-to-liver (T/L) ratios were significantly improved in the pretargeting approach: 7.8 vs. 4.2 and 2.8 vs. 0.8, respectively (29).

Besides using a pretargeting approach to detect tumor lesions, the pretargeting approach was also tested to treat tumor lesions. A clinical trial was conducted to estimate the dose delivered to tumor targets and normal tissues using a pretargeting approach consisting of bsAb anti-CEA × anti-DTPA and ^131^I-diDTPA (30). In the clinical trial patients with recurrent medullary thyroid carcinoma (MTC; five patients) and small-cell lung cancer (SCLC; five patients) were pretargeted with a bsAb anti-CEA × anti-DTPA Fab′–Fab′ (0.1–0.3 mg/kg) and 4 days later 6 nmol (5.8–9.8 mCi) of ^131^I-di-DTPA was administrated. The patients were included based on the CEA expression as evaluated by immunohistochemical analysis of the biopsy of their primary tumor. The tumor uptake of ^131^I-di-DTPA and the activity dose to the tumor was significant higher in MTC (average uptake 0.116%ID/g; dose ranged from 4.8 to 135 cGy/mCi) than in SCLC (average uptake 0.009%ID/g; dose ranged from 1.9 to 8 cGy/mCi), indicating that the MTC is a more suitable tumor type for PRIT. The therapeutic efficacy and toxicity of PRIT with escalating doses of bsAb anti-CEA × anti-DTPA (20–50 mg) and 4 days later followed by ^131^I-di-DTPA (1.48–3.7 GBq) was evaluated in 26 patients with recurrent MTC (24). The dose-limiting toxicity was hematologic and an activity dose of 1.78 GBq/m^2^ could be injected safely in this group of patients. The maximum tumor uptake ranged from 0.003 to 0.26%ID/g and the tumor doses ranged from 7.9 to 500 Gy/MBq (2.94–184 cGy/mCi). From the 26 patients, 17 patients were included to evaluate the tumor response. The maximal tolerated dose (MTD) of 48 mCi/m^2^ was administrated to 13 patients. A decrease in tumor mass, serum thyrocalcitonin, and CEA levels was observed in six of the patients receiving the MTD. The tumors that showed a response were generally small (maximum diameter of 37 mm).

Despite the promising (pre)clinical outcomes using a divalent hapten-peptide, the antibody dissociation rates were still higher as compared to the high affinity pretargeted avidin–biotin complex (*K*_d_ = 10^−14^ M for biotin with streptavidin/avidin vs. 10^−9^ M for most antigen–antibody complexes) (7). It was suggested that bsAb divalency to the primary target antigen might result in higher tumor accretion by the pretargeted divalent peptide (21). A bsAb chemically prepared by coupling two Fab′ fragments, one with monovalent specificity to CEA and one to ^111^In-DTPA (Fab′ × Fab′) was compared to bsAb with divalency to CEA. Two divalent CEA bsAb coupled to a DTPA Fab′fragment were composed either of the whole anti-CEA IgG (IgG × Fab′) or the anti-CEA F(ab′)_2_ fragment [F(ab′)_2_ × Fab′). The antibody constructs differ in molecular weight resulting in different clearance rates from the blood of which the fastest clearance is observed with lowest molecular weight construct, Fab′ × Fab′, followed by F(ab′)_2_ × Fab′and slowest for IgG × Fab′. The highest uptake and retention in the tumor was achieved using the IgG × Fab′ bsAb conjugate. However, in a pretargeting strategy, the uptake of the ^111^In-di-DTPA peptide (^111^In-IMP-192) was highest using F(ab′)_2_ × Fab′conjugate followed by Fab′ × Fab′ conjugate and lowest for IgG × Fab′. At the time of maximum tumor accretion, the IgG × Fab′ in the blood predominantly captured the injected hapten. As a consequence, the three- to fourfold higher tumor accumulation of the IgG × Fab′ could not be exploited in the pretargeting approach. Consequently, to take full advantage of a high uptake and retention in the tumor using a long circulating bsAb, the accessibility of the bsAb remaining in the blood need to be reduced by using a clearing agent (21).

The flexibility of the bsAb pretargeting system is increased using the second generation anti-chelate bsAb as different radionuclides can be used, for example, ^111^In (21, 29) for imaging and ^131^I for treatment (24, 30). However, the binding affinity can be affected by the chelated metal. For example, the affinity of the anti-DTPA antibody, designated 734, to various radiometal labeled DTPA varies from 10^−9^ M for In-DTPA to 10^−3^ M for Ca-DTPA (31). Additionally, the anti-(In)DTPA had a much lower affinity for DTPA with other radiometals such as ^90^Y or ^177^Lu. Therefore, it appeared that imaging radionuclides require different antibodies directed against the specific anti-chelate–metal complex than the radionuclides of therapeutic interest.

### Third generation; bispecific F(ab′)2 anti-CEA × anti-histamine-succinyl-glycine

A more universal/flexible pretargeting system was developed by using an anti-hapten antibody not directed against the chelated radiometal, but directed against the histamine-succinyl-glycine (HSG) hapten (22, 32, 33). The HSG hapten itself is not involved in binding the radionuclide and thus other chelates suitable for binding radionuclides can be added, for example, the HSG peptides can be conjugated with various chelating moieties such as DTPA, DOTA, or N3S chelates for radiolabeling with In-111, Lu-177/Y-90, or Tc-99m/Re-188, respectively or with other agents/moieties such as fluorophores for fluorescence imaging. Sharkey et al. showed the potential of this system by using the bispecific anti-CEA F(ab′)_2_ × anti-HSG Fab in combination with HSG peptides labeled with either ^99m^Tc or ^188^Re via an N3S chelate or with ^111^In, ^177^Lu, and ^90^Y via a DOTA moiety (32). In nude mice with human colon tumor xenografts, this trivalent anti-CEA × anti-HSG bsAb was injected 24–48 h prior to the i.v. administration of the divalent radiolabeled HSG peptides, ^111^In-IMP241, ^177^Lu-IMP241, and ^99m^Tc-IMP243. The tumor uptake of radiolabeled HSG peptides after pretargeting with the bsAb was significantly higher than with the peptides alone (for ^111^In-IMP241 0.03 ± 0.02 vs. 2.54 ± 1.04%ID/g, for ^177^Lu-IMP241 0.03 ± 0.01 vs. 2.59 ± 0.3%ID/g, and for ^99m^Tc-IMP243 0.1 ± 0.02 vs. 7.36 ± 3.19%ID/g). The tumor-to-normal tissue ratio exceeded 8:1 within 3 h p.i. of the hapten-peptide. Consequently, this provides the opportunity to use the same HSG peptide for different purposes depending on the radionuclide used, for example, using ^111^In and ^99m^Tc-labeled HSG peptide for SPECT imaging, ^124^I, ^68^Ga, or ^18^F-labeled HSG peptide for PET imaging or ^131^I, ^90^Y, and ^177^Lu-labeled HSG peptide for PRIT (32–38). Besides the flexibility of the system, Sharkey et al. showed that the hapten–peptide structure can alter the biodistribution and clearance of the construct. The liver and kidneys uptake of ^99m^Tc-hapten-peptide consisting of a N3S chelate and DOTA (IMP245) was significantly lower compared to the ^99m^Tc-hapten-peptide bearing only the N3S chelate whereas the tumor uptake and retention did not change. Therefore, when less background is essential around the kidneys and/or urinary bladder, the preferable renal elimination can be altered to a more hepatic elimination by modifying the hapten-peptide construct.

Further improvements were obtained using a recombinant bispecific trivalent construct, hBS14, with bivalent CEA binding and a monovalent HSG binding (39). The trivalent antibody was produced by myeloma cells transfected with hBS14-pdHL2 DNA vector and purified to near homogeneity in a single step using a novel HSG-based affinity chromatography system. In nude mice bearing a CEA-expressing GW-39 human colon tumor xenograft the efficacy of the ^125^I-hBS14 (0.4 nmol) in combination with the bivalent ^111^In-HSG-hapten (0.04 nmol; 27 h interval), IMP241, was evaluated. At 3 h p.i., the ^111^In-IMP241 tumor uptake was 19.1 ± 8.7% ID/g and the blood levels were only 0.25 ± 0.08% ID/g corresponding with a tumor-to-blood ratio of 83 ± 44% ID/g.

**Figure 3:**
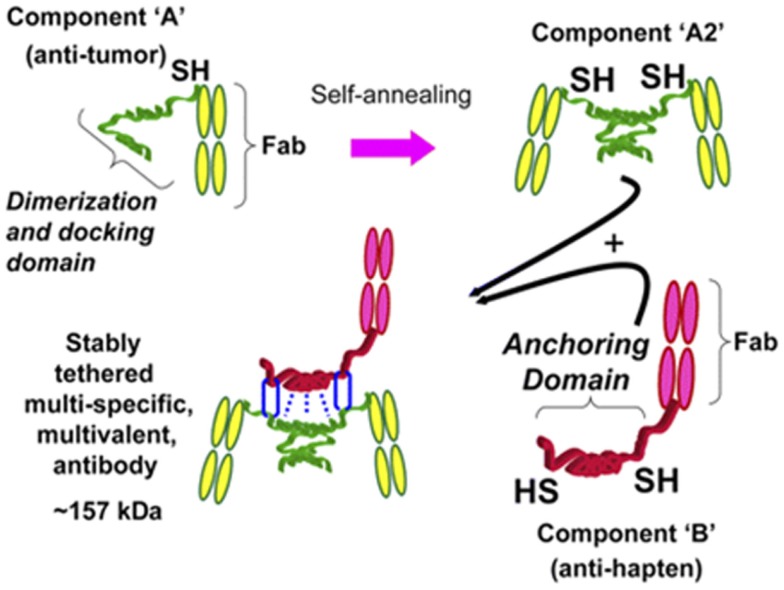
**Schematic representation of how a trivalent bispecific antibody is formed by the DNL method**. Component A links a cysteine-modified dimerization and docking domain (DDD) to a Fab of an anti-tumor antibody, which results in spontaneous formation of component A2. Component B links an anchoring domain to a Fab of an anti-hapten antibody. The AD is modified with cysteine on each end. AD will naturally “dock” with the DDD when components A and B are mixed, which brings the two molecules together in a well-defined orientation and also results in disulfide bonds across the two proteins. This research was originally published in Goldenberg et al. (42).

Technology that exploits fusing two hybridoma Ab-producing cells using quadroma technology did not produce adequate yields in mammalian cell cultures. Therefore, a new strategy capable of preparing a trivalent bsAb was developed. Humanized trivalent Fab bsAb constructs were produced at a higher yield by the dock-and-lock (DNL) technology. The DNL technology is based on the natural association between protein kinase A (PKA; cyclic AMP-dependent protein kinase) and A-kinase anchoring proteins. The regulatory subunit of PKA and the anchoring domain of the interactive A-kinase anchoring protein are both attached to a biological entity. The dimeric sequence attached to the anti-tumor F(ab)_2_ has high affinity for the sequence attached to the anti-hapten Fab, the so-called docking. The stabilization of the trivalent bispecific antibody construct, the so-called locking, is secured by the placement of cysteine residues on four locations within the construct resulting in the formation of disulfide bridges (40–42). A schematic overview of the DNL technology is shown in Figure 3.

Using the DNL technology, several recombinant humanized bsAbs with a divalent antigen specificity and with a monovalent specificity to HSG were produced including an anti-CEA bsAb (TF2) (43, 44), an anti-Trop2 (TF12) (45), an anti-CD20 (46), and an anti-MUC1 (47) bsAb.

An effective detection and treatment of tumor lesions using a pretargeting system is best illustrated by the work done on TF2. The ability to detect small micrometastatic human colon cancer nodels (<0.3 mm in diameter) in the lungs of nude mice using this system was evaluated and compared to ^18^F-FDG-PET imaging and to TF6, an irrelevant anti-CD22-based bsAb. The tumors were first pretargeted with TF2 and 21–24 h later the ^124^I or ^131^I radiolabeled hapten-peptides were injected at a bsAb:hapten molar ratio of 10:1. The TF2-pretargeted tumors in the lungs could be localized at 1.5 h p.i. of radiolabeled HSG peptide whereas the peptide alone, TF6, or ^18^F-FDG failed. This showed the high potential of the system for sensitive and specific imaging of tumor lesions.

**Figure 4:**
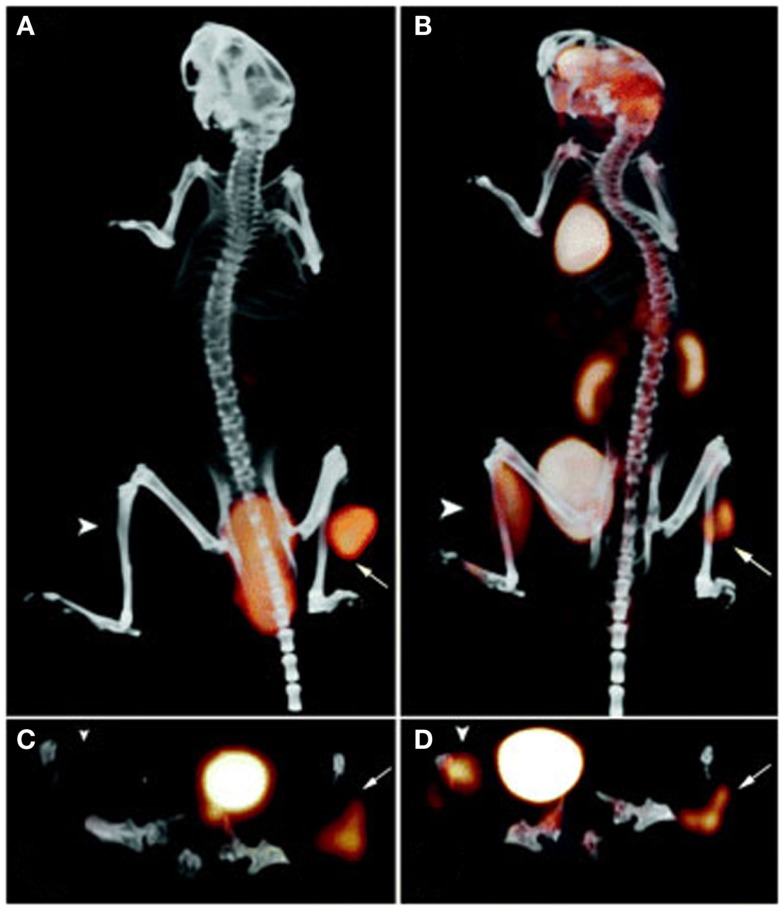
**PET/CT images of a BALB/c nude mouse with a s.c**. LS174T tumor on the right hind leg (arrow) and an inflammation in the left thigh muscle (arrowhead), which received ^18^F-FDG and, 1 day later, 6.0 nmol TF2 and ^68^Ga-IMP288 (0.25 nmol) with a 16-h interval. The animal was imaged 1 h after ^18^F-FDG and ^68^Ga-IMP288 injections. The panel shows the three-dimensional volume rendering the pretargeted immuno-PET scan **(A)** and the FDG-PET scan **(B)**, and the transverse sections of the tumor region of the pretargeted immuno-PET scan **(C)** and the FDG-PET scan **(D)**. This research was originally published in Schoffelen et al. (48, 50).

The specificity of the TF2 based pretargeting system was further evaluated using di-HSG peptides radiolabeled with ^68^Ga, a positron emitting radionuclide with a more suitable half-life (68 min) for pretargeted imaging purposes than ^124^I (4.2 days) (48). Nude mice were s.c. implanted with CEA-expressing LS174T human colonic tumor, a CEA-negative tumor, or an inflammation was induced in the thigh muscle. The mice were first i.v. injected with bsAb anti-CEA × anti-HSG and after 16 h followed by ^68^Ga-IMP288. At 1 h p.i., a high specific uptake of ^68^Ga-IMP288 was observed in the tumor (10.7 ± 3.6%ID/g), whereas the uptake of ^68^Ga-IMP288 in normal tissues, in CEA-negative tumors and inflamed muscle was low (Figure 4). In contrast, ^18^F-FDG localized efficiently in the tumor, however, also in the inflamed tissue and in a number of normal tissues including liver, spleen, and intestines. The specificity of the pretargeted immuno-PET using TF2 and ^68^Ga-IMP288 was confirmed in mice with small intraperitoneal xenografts (49). All intra-abdominal tumors lesions >~4 mm and even a few lesions as small as ~2 mm were detected. In line with the previous study, the ^18^F-FDG uptake in all the present tumors was sufficient, however, also an uptake was observed in various normal tissues such as the intestines.

To evaluate the use of the TF2 and ^177^Lu-IMP288 in PRIT, the therapeutic efficacy and toxicity of the system was evaluated in mice with s.c. LS174T tumors (50). The median survival of mice treated with 1, 2, or 3 cycles of PRIT was 24, 45, and 65 days, respectively, whereas in the untreated mice the median survival was 13 days. Additionally, PRIT effectively delayed the tumor growth with limited hematologic toxicity, indicating that PRIT using TF2 and ^177^Lu-IMP288 might be an effective treatment against colon cancer. Furthermore, the biodistribution of ^111^In-IMP288 and ^177^Lu-IMP288 in mice with intraperitoneal LS174T tumors was identical as observed by *ex vivo* counting as well as by pretargeted immuno-SPECT imaging (51). This indicates that pretargeted immuno-SPECT with TF2 and ^111^In-IMP288 can be used for the non-invasive monitoring of the therapeutic efficacy of PRIT with TF2 and ^177^Lu-IMP288 in mice with LS174T lesions.

**Figure 5:**
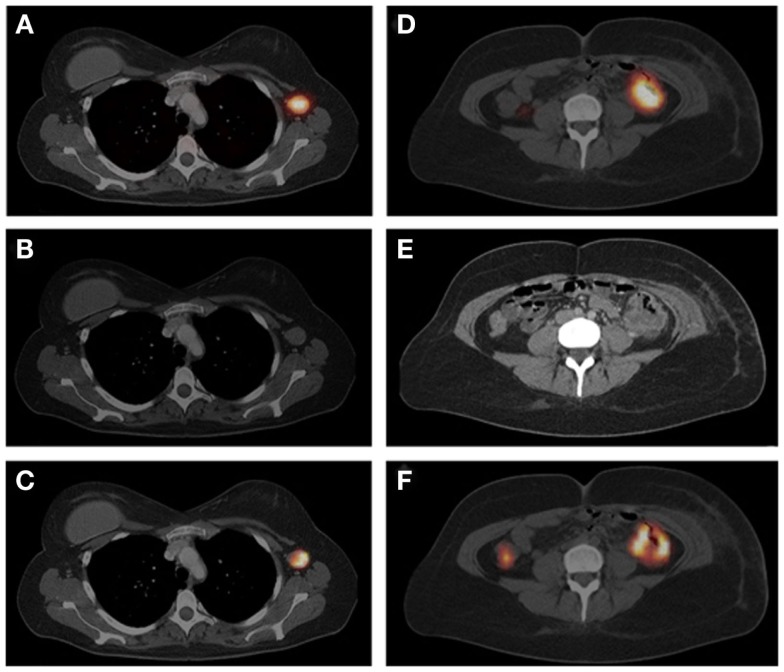
**The SPECT/CT image (A), acquired 24 h after injection of ^111^In-IMP288 (185 MBq, 25 μg), pretargeted with 75 mg TF2 (1-day interval), in a 38-year-old patient (cohort 4), shows very clear tumor targeting of an axillary lymph-node metastasis, with very low concentrations of radioactivity in normal tissues**. Corresponding contrast-enhanced CT scan and a fused FDG-PET/CT scan are shown [**(B,C)**, respectively]. The primary colon tumor (50 cm ab ano) also shows highly specific tumor targeting in the SPECT image **(D)**, confirmed by the CT scan and FDG-PET/CT [**(E,F)**, respectively], with non-specific FDG uptake in the ascending colon. This research was originally published in Schoffelen et al. (52).

The preclinical pretargeting studies using TF2 in combination with radiolabeled di-HSG peptides indicated that PRIT can induce tumor growth inhibition. Therefore in the feasibility, safety and therapeutic efficacy of TF2/Lu-177-IMP288 for the treatment of CEA-expressing tumor lesions was investigated in a first-in-man phase I study in patients with advanced colorectal carcinoma. Four dose schedules in cohorts of five patients were evaluated (52). First, the effect of the time interval between administration of TF2 (75 mg) and IMP288 (100 μg) was evaluated: 5 days (cohort 1) and 1 day (cohort 2). Additionally, the effect of a higher bsAb dose (150 mg TF2, 1-day interval, 100 μg IMP288, cohort 3) and a lower IMP288 dose (75 mg TF2, 1-day interval, 25 μg IMP288, cohort 4) was evaluated. Reducing the time interval and lowering the IMP288 dose resulted in improved tumor targeting. Although it is reported that higher bsAb doses results in improved tumor uptake of the radiolabeled hapten (48, 50), no such observations were found using twofold increase of TF2 dose. Co-localization of almost all ^18^F-FDG positive tumors and hapten-peptide with SPECT was observed (Figure 5). Using a high TF2 dose and a low IMP288 peptide dose rapid targeting of tumors was observed, although wash-out of the tumor was observed after 24 h. The patients could tolerate 7.4 GBq of ^177^Lu-IMP288 without experiencing dose-limited toxicity, even though some patients (8 out of 20 patients) experienced some level of hematologic toxicity. The TF2 bsAb induced human anti-human antibodies (HAHA) in 11 out of 21 patients. Most likely, the best PRIT regime will be a fractionated multi-dose treatment regimen (e.g., multiple cycles) as observed in preclinical pretargeting experiments (50) and recent clinical trials using ^90^Y-labeled antibodies in combination with gemcitabine (53). However, further clinical studies regarding the pretargeting conditions and protocol are needed.

## A Different Approach; Pretargeting Based on Bioorthogonal Chemistry

**Figure 6:**
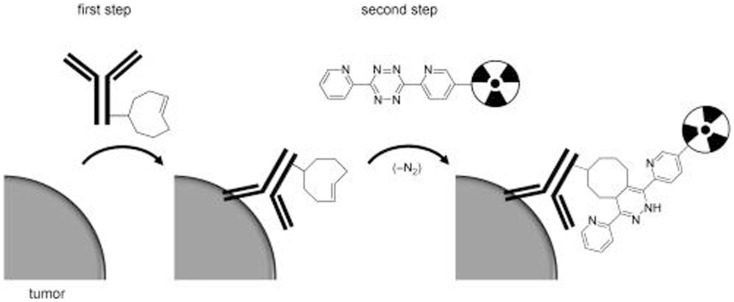
**Schematic overview of tumor pretargeting by using the inverse-electron-demand Diels–Alder reaction**. This research was originally published in Rossin et al. (60).

**Figure 7:**
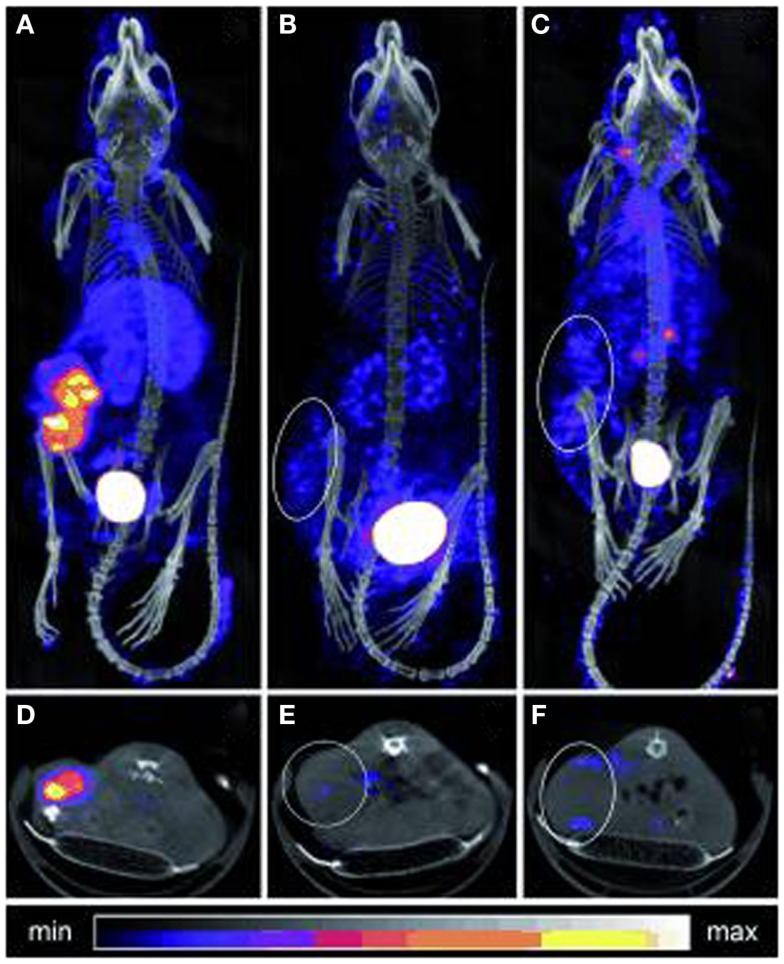
**SPECT/CT imaging of mice bearing colon carcinoma xenografts: posterior projections of mice preinjected with (A) CC49-TCO followed 1 day later by ^111^In-DOTA-tetrazine (1:25, 42 MBq), (B) CC49 followed 1 day later by ^111^In-DOTA-tetrazine (1:25, 20 MBq), (C) irrelevant Ab (Rtx-TCO; 100 μg) followed 1 day later by ^111^In-DOTA-tetrazine (1:25, 50 MBq), (D–F) single transverse slices (2 mm) passing through the tumors in** **(A–C)**. This research was originally published in Rossin et al. (60).

The combination of chemistry and biology has resulted in many innovations and has contributed to our understanding of many biological processes. Nevertheless, many biomolecules including lipids, glycans, and nucleic acids as well as various posttranslational modifications cannot be monitored with genetically encoded reporters as manipulations can interfere with the structure and function of the molecules or the molecules are not genetically encoded. Therefore, new methods were investigated to covalently modify biomolecules *in vivo*. This has led to the discovery of bioorthogonal reactions to detect, track, or visualize various biomolecules. Using bioorthogonal chemistry, as defined by Bertozzi et al., reactions can occur inside a living organism, which do not interfere or cross-react with naturally occurring functionalities, are reactive under mild and physiological conditions and should not induce cellular toxicity (54). Over the years, several bioorthogonal chemical ligation strategies have been developed including the tetrazine ligation, Staudinger ligation, oxime/hydrazone formation from aldehydes and ketones, and quadricyclane ligation [for reviews on these bioorthogonal chemical reactions see in Ref. (13, 55, 56)]. The archetypical bioorthogonal reaction is the copper-catalyzed 1,3-dipolar Huisgen “click” cycloaddition between azides and alkynes (55). Using this click chemistry, units are joined with heteroatom links (C-X-C) in a modular, rapid, and easy manner without the generation of toxic byproducts (57). The selectivity and flexibility as well as the bioorthogonality and speed of click chemistry make it an ideal method for the creation of radiopharmaceuticals, especially ^18^F-radiolabeled peptides (58). The binding of a radiolabeled probe to the tumor-targeted antibody might also be obtained using bioorthogonal reactions. In general, this chemical approach of pretargeting is less likely to be immunogenic and could provide a universal approach for tagging and *in vivo* tracking of Abs. However, the need for a copper catalyst in coupling of azide and terminal alkyne to generate a triazole limits the use of the 1,3-Huisgen cycloaddition reaction in biological systems due to the toxicity of copper *in vivo*. Various bioorthogonal reactions have been developed that do not need the presence of a Cu catalyst. For example, copper-free click chemistry can be achieved via the relief of strain (strain-promoted azide-alkyne cycloadditions, SPAAC). Alternatively, Blackman et al. reported the very fast reaction kinetics and *in vitro* bioorthogonality of the inverse-electron-demand Diels–Alder (IEDDA) reaction between *trans-*cyclooctene (TCO) and electron deficient tetrazines (59). Subsequently, Devaraj et al. tagged the anti-EGFR antibody cetuximab with *trans*-cyclooctene succinimidyl carbonate and combined with a fluorescent tetrazine probe was able to target A549 cancer cells in serum at 37°C (60). A schematic overview depicting the use of TCO-modified mAb and radiolabeled tetrazine to (pre)target tumors through the IEDDA reaction is shown in Figure 6. The first *in vivo* proof-of-concept study by Rossin et al. using a chemical pretargeting approach based on the Diels–Alder components demonstrated that the system could manage the more demanding conditions *in vivo*, including low reagent concentrations and short reaction time, and the prolonged residence time and required *in vivo* stability for the TCO tag (61). In mice bearing a colon-cancer xenografts, TCO-modified anti-TAG72 mAb CC49 (CC49-TCO) was administrated and 1 day later followed by ^111^In-labeled-DOTA-tetrazine (^111^In-tetrazine) at a 1:25 molar ratio. Three hours p.i. of ^111^In-tetrazine, SPECT imaging clearly delineated the tumor with a tumor uptake of 4.2%ID/g and a tumor-to-muscle (T/M) ratio of 13.1, whereas the tumor uptake of ^111^In-tetrazine and tumor-to-muscle ratio pretargeted with unmodified CC49 or an irrelevant TCO-modified Ab were 0.3 and 0.5%ID/g or 1.0 and 2.1%ID/g, respectively (Figure 7).

**Figure 8:**
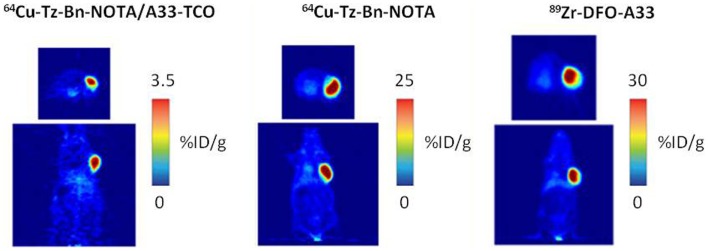
**PET images of 64Cu-Tz-Bn-NOTA/A33-TCO pretargeting strategy, 64Cu-NOTA-A33 and 89Zr-DFO-A33**.Transverse (top) and coronal (bottom) planar images intersect the center of the tumors. This research was originally published in Zeglis et al. (63).

Zeglis et al. demonstrated that the *in vivo* click methodology is able to delineate tumors with PET (62). Nude mice with s.c. SW1222 colorectal cancer xenografts were i.v. administrated with TCO-modified A33 Ab (100 μg) followed 24 h later by ^64^Cu-NOTA-tetrazine (10.2–12.0 MBq) or with the directly radiolabeled Ab ^64^Cu-NOTA-A33 (10.2–12 MBq) or ^89^Zr-DFO-A33 (10.2–12.0 MBq). Despite the higher tumor accumulation of the directly labeled antibodies at 24 h p.i, the pretargeting approach resulted in comparable PET images and tumor-to-muscle ratios (Figure 8).The dose delivered to normal tissues using this pretargeting approach was calculated, indicating that the non-targeted tissues received a significant lower dose when a pretargeting approach (0.0124 mSv/MBq) was used compared to the directly labeled Abs, ^64^Cu-NOTA-A33, and ^89^Zr-DFO-A33 (0.0359 and 0.4162 mSv/MBq, respectively).

The fast reaction kinetics [*k_2_* = 13,090 M^−1^s^−1^ (60)] of the IEDDA reaction between the trans-cycloctene and tetrazines are very promising, however, are still significantly lower compared to the association constants of the non-covalent high affinity interactions used in humans (5 × 10^5^ up to 7.5 × 10^7^ M^−1^s^−1^). In addition, Rossin et al. reported that the TCO can be deactivated through isomerization to the unreactive *cis*-cyclooctene (CCO) isomer via copper-containing proteins (e.g., transcuprein, mouse serum albumin, ceruloplasmin) (61). TCO tags that are linked to the antibody through an axial substituent afforded a marked increase of the reactivity (61). In conjunction, the stability of the TCO tag could be improved by removal of the PEG linker between the TCO and the lysine residue on the Ab, increasing the steric hindrance on the TCO thus hampering interaction with serum protein-bound copper. The increased reactivity (up to *k_2_* = 2.7 × 10^5^ M^−1^s^−1^ in PBS (61) and improved stability of the IEDDA between a tetrazine and TCO-tagged antibody resulted in an improved tumor-to-blood ratio (61). Devaraj et al. (64) reported that the *in vivo* reaction yield of a moderately reactive IEDDA system (*k_2_* = 6 × 10^3^ M^−1^s^−1^ in PBS) could be improved by altering the pharmacokinetics of the radiolabeled probe by polymer conjugation (64). In mice with LS174T xenografts TCO-tagged A33 antibody (30 μg) was administrated and followed 24 h later by the ^18^F-labeled, dextran-based tetrazine probe (30 μg; 150 μCi). PET imaging 3 h p.i. of the ^18^F-labeled tracer revealed a significant higher tumor accumulation in TCO-tagged antibody pretargeted mice compared to mice pretargeted with Ab lacking TCO. However, the tumor-to-blood ratio still remained low due to the relative high amount of free-circulating CC49-TCO. For effective tumor targeting, a clearing agent was developed that could clear the TCO-tagged antibody from the circulation prior to administration of the radiolabeled tetrazine (61). In mice with LS174T tumor xenografts, ^125^I-CC49-TCO was injected after which a single or double dose (30 h or 30 and 48 h after mAb injection) of the clearing agent, followed 2 h later by ^177^Lu-tetrazine. Three hours p.i., the blood level of ^125^I-CC49-TCO was lowest after the double dose of clearing agent (0.19 ± 0.04%ID/g), followed by the single dose (1.16 ± 0.43%ID/g) and highest in the group without clearing agent injection (8.47 ± 4.12%ID/g). The biodistribution of ^177^Lu-tetrazine showed that the elimination of the free CC49-TCO in combination with a reduced amount of tetrazine (from 17 to 6.7 nmol) significantly improved the tumor uptake from 3.12 ± 0.87%ID/g (60) to 7.45 ± 1.46%ID/g for the single dose and to 6.13 ± 1.09%ID/g for the double dose of clearing agent. Both single as well as the double dose approach significantly increased the tumor-to-muscle and tumor-to-blood ratios compared to the previous approach without clearing agent. In fact, a 125-fold improvement of the tumor-to-blood ratio at 3 h after tetrazine injection was achieved with a double dose of clearing agent. Corresponding mouse dosimetry experiments suggested that at MTD for bone marrow (dose-limiting organ for both approaches), this should allow for an eightfold higher tumor dose than is possible with non-pretargeted RIT. The estimated dose to the tumor in mice with directly ^177^Lu-labeled CC49 is significantly higher compared to the pretargeted approach using CC49-TCO and ^177^Lu-Tz (9573 vs. 479 mGy/MBq). However, also the normal tissues receive high doses, for example, the bone marrow receive 1136 mGy/MBq, whereas in the pretargeting approach the bone marrow dose is 7 mGy/MBq. Subsequently, this system was further improved by modifying the pharmacokinetics of the tagged antibody and by increasing the stability of the tag. The use of a more hydrophilic tag with an increased *in vivo* tag stability (*t*_1/2_ = 10 days) afforded a slower clearing CC49-TCO conjugate with increased tumor targeting, resulting in a 50% increased tumor uptake and T/NT ratios of the tetrazine probe (65). So far, the reaction of TCO with 1,2,4,5-tetrazines is the fastest bioorthogonal reaction (61). While others were unsuccessful in enlisting the slower SPAAC for antibody-based pretargeting in mice (62), Lee et al. reported a pretargeting system consisting of aza-dibenzocyclooctynes modified mesoporous silica nanoparticles in combination with ^18^F-azide and achieved tumor uptake in mice, possibly due to the high loading capacity of the mesoporous particles (66). Further work is required to establish whether the fastest cyclooctynes enable efficient SPAAC-based pretargeting with antibodies as well.

## Conclusion

Clinical studies revealed that the pretargeting approach based on bsAb directed against a tumor-associated antigen and against HSG in combination with radiolabeled (divalent) peptides containing the HSG residue resulted in high contrast images. Therefore, the use of this pretargeting system is very promising for non-invasive imaging of tumors before, during, and after therapy. Additionally, pretargeted RIT using this system is capable to inhibit tumor growth with minor toxicity. However, to fully develop this pretargeting approach in the clinic research should focus on the best regime and protocol. Although several comparative studies between a pretargeting approach and directly radiolabeled antibodies have been performed, further quantitative comparative studies are needed to evaluate the potential of the pretargeted imaging and therapy. Furthermore, preclinical studies using bioorthogonal chemical pretargeting based on the inverse Diels–Alder cycloaddition revealed that tumors can be imaged and treated with this new approach. This approach is still being further optimized and clinical studies are warranted to determine the potential of this new strategy.

## Conflict of Interest Statement

The authors declare that the research was conducted in the absence of any commercial or financial relationships that could be construed as a potential conflict of interest.
